# Case Report: Long-term survival in advanced PD-L1-high squamous cell lung cancer following severe immune-related cardiotoxicity

**DOI:** 10.3389/fimmu.2026.1777957

**Published:** 2026-05-01

**Authors:** Qi-Qing Zhang, Xin-Xin Luo, Hua Chen, Wei Wang, Jun Liu, Ying Li, Hong-Li Qiao, Lang Chen, Zheng-Quan Feng

**Affiliations:** 1 Department of Oncology, Tongde Hospital of Zhejiang Province, Hangzhou, Zhejiang, China; 2 Department of Geriatrics, Yuyao Maternity and Child Health Care Hospital (Yuyao Second People’s Hospital), Ningbo, Zhejiang, China; 3 College of Integrated Traditional Chinese and Western Medicine Clinical Medicine, Zhejiang Chinese Medical University, Hangzhou, Zhejiang, China; 4 Department of Pathology, Tongde Hospital of Zhejiang Province, Hangzhou, China; 5 Department of Pharmacy, Tongde Hospital of Zhejiang Province, Hangzhou, China; 6 Department of Endocrinology, Tongde Hospital of Zhejiang Province, Hangzhou, China

**Keywords:** immune-related cardiotoxicity, immunotherapy, non-small cell lung cancer, PD-L1-high, squamous cell carcinoma

## Abstract

The introduction of immune checkpoint inhibitors (ICIs) has reshaped the therapeutic landscape of advanced non-small cell lung cancer. However, immune-related adverse events (irAEs), including rare cases of life-threatening cardiotoxicity, pose a significant challenge for subsequent therapy selection. Evidence on optimal post-ICI strategies, particularly in patients who have experienced severe irAEs, remains limited. Herein, we report the case of a 58-year-old male smoker with stage IVA squamous cell lung carcinoma (cT4N3M1a, PD-L1 TPS 60%, EGFR wild-type) who developed grade 3 immune-mediated myocarditis after receiving chemoimmunotherapy, leading to permanent ICI discontinuation. After improvement of the immune-related myocarditis, the patient was administered second- and third-line treatment. The patient achieved a progression-free survival of 31 months following third-line treatment, with an overall survival of 43 months. This case demonstrated successful reversal of severe immune-related cardiac toxicity in a patient with advanced squamous cell lung cancer with high PD-L1 expression through personalized, precise, and multidisciplinary team diagnosis and treatment, achieving prolonged overall survival. Furthermore, this case suggested that anti-angiogenic therapy (anlotinib) can be safely administered as subsequent treatment for lung squamous cell carcinoma following recovery from severe immune-related cardiotoxicity.

## 1 Introduction

Lung cancer is the deadliest cancer worldwide, accounting for 18% of all cancer-related deaths (1). Among these, non-small cell lung cancer (NSCLC) accounts for approximately 85% of all lung cancers and has a relatively high mortality rate. Surgical resection remains the best treatment option for patients with lung cancer. In addition to traditional chemotherapy and radiotherapy, the emergence of immunotherapy and targeted therapy has greatly improved clinical outcomes in patients with lung cancer (2). As immune checkpoint inhibitors (ICIs) are increasingly applied in cancer treatment, many cancer patients derive significant benefit. Integrating PD-1/PD-L1 inhibitors into first-line treatment for advanced NSCLC has improved survival outcomes, particularly in patients with high PD-L1 expression (3–5). Unlike standard chemotherapy or other biologics, ICI-related toxicity primarily stems from excessive immune reactions against normal tissues (6).

ICIs and their combinations can cause various immune-related toxicities, including cardiotoxicity, colitis, hepatitis, pneumonitis, thyroiditis, myositis, hypophysitis, and dermatitis (7). These immune-mediated adverse effects are generally reversible and manageable with glucocorticoid therapy (8). However, immune-related cardiotoxicity, although rare (incidence <1%) (9), can have a considerable clinical impact, with mortality rates reaching 25–50% (8). ICI-related cardiac toxicity is rare, with myocarditis being the most common manifestation, accounting for approximately 50.8% of cases. Other cardiac immune-related adverse events (irAEs) include arrhythmia, myocardial fibrosis, pericarditis, acute heart failure, and cardiomyopathy with Takotsubo-like syndrome (10–12).

A large retrospective survey reported a myocarditis incidence rate of 1.14%. The incidences of myocarditis in patients treated with PD-1, PD-L1, and cytotoxic T lymphocyte-associated protein 4 (CTLA-4) inhibitors alone are 0.5%, 2.4%, and 3.3%, respectively. Conversely, the incidence rates in patients treated with PD-1 + CTLA-4 and PD-L1 + CTLA-4 inhibitor combinations are 2.4% and 1%, respectively (13). However, the real-world incidence may be underestimated.

Current guidelines recommend permanent discontinuation of ICIs in patients who develop grade 3–4 cardiotoxicity; however, evidence regarding optimal subsequent therapy remains scarce (14). Herein, we present a case of grade 3 cardiotoxicity after initial chemoimmunotherapy in squamous NSCLC with high PD-L1 expression, in which salvage anti-angiogenic therapy with anlotinib achieved disease control for over 31 months. Although the patient ultimately died from a pulmonary infection, this case highlights the possibility of successful reversal of severe immune-related cardiotoxicity in advanced squamous cell lung cancer with high PD-L1 expression, achieving prolonged progression-free survival (PFS) and overall survival (OS). Thus, this report provides a clinical reference for the management of immune-related adverse reactions.

## 2 Case presentation

### 2.1 Diagnosis

**Figure 1:**
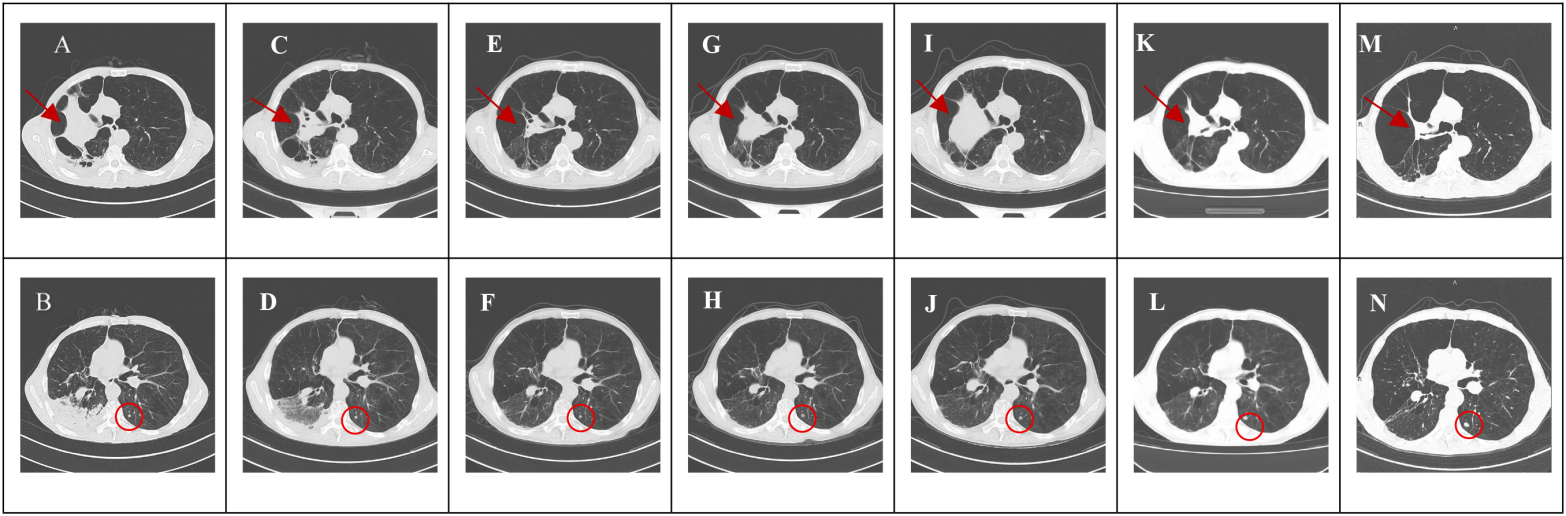
Chest CT scan. Initial chest CT (May 12, 2022). **(A)** Right upper lobe mass (arrow) with obstructive inflammation, atelectasis, suspected mediastinal and right axillary lymphadenopathy, and bronchial occlusion. **(B)** Ill-defined nodule in the left lung (circle). Chest CT after 1 cycle of immunotherapy and chemotherapy (June 18, 2022). **(C)** The right upper lobe mass has reduced in size (arrow). **(D)** The ill-defined nodule in the left lung is stable in size (circle). Chest CT after 5 cycles of chemotherapy (October 27, 2022). **(E)** The right upper lobe mass has reduced in size (arrow). **(F)** The ill-defined nodule in the left lung is stable in size (circle). Chest CT after progression on first-line treatment (January 14, 2023). **(G)** The right upper lobe mass has increased in size (arrow). **(H)** The ill-defined nodule in the left lung is stable in size (circle). Chest CT after progression on second-line treatment (March 6, 2023). **(I)** The right upper lobe mass has markedly increased in size (arrow). **(J)** The ill-defined nodule in the left lung is stable in size (circle). Chest CT two months after third-line treatment (June 8, 2023). **(K)** The right upper lobe mass has markedly reduced in size (arrow). **(L)** The ill-defined nodule in the left lung is stable in size (circle). Chest CT thirty-one months after third-line treatment (November 7, 2025). **(M)** The right upper lobe mass remains stable (arrow). **(N)** The ill-defined nodule in the left lung is stable in size (circle).

**Figure 2:**
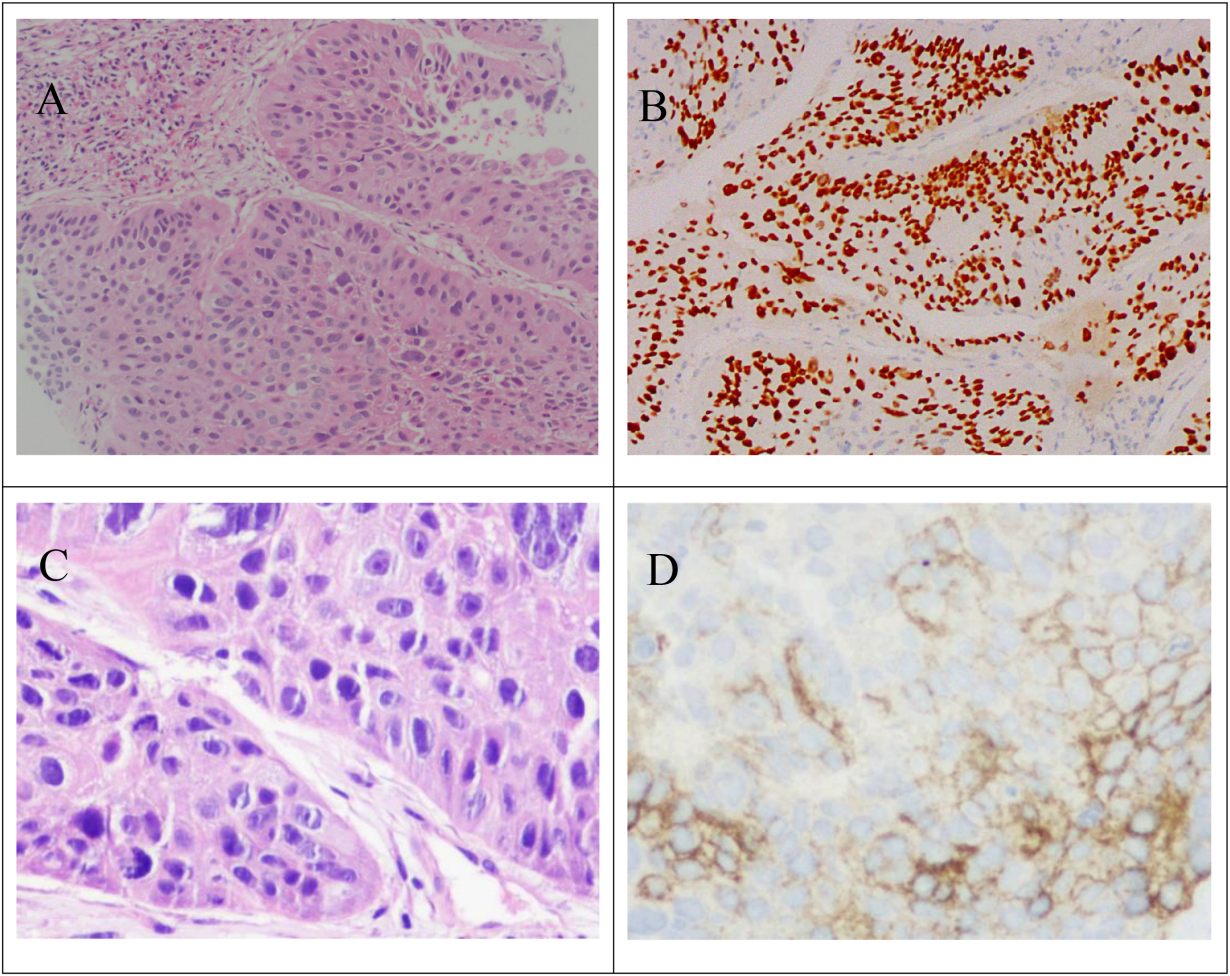
Pathological examination of the lung biopsy sample. **(A)** HE staining (100×) shows nested tumor cells with keratin pearl formation. **(B)** P40 staining (100×) reveals diffuse nuclear positivity, confirming squamous differentiation. **(C)** HE staining (200×) highlights intercellular bridges and atypical mitotic figures. **(D)** PD-L1 staining (200×) demonstrates membranous expression on tumor cells.

In April 2022, a 58-year-old male (168 cm, 46 kg) who presented with “cough with sputum and fever” was admitted to the cardiothoracic surgery Tongde Hospital of Zhejiang Province. Laboratory tests after admission indicated elevated tumor markers. He was transferred to the oncology department for further treatment after two weeks, where a chest CT scan revealed a mass in the right upper lobe. The lesion showed obstructive inflammation and atelectasis, with suspected lymph node metastases in the mediastinum and right axilla, and occlusion of the right upper lobe bronchus, suggestive of malignancy (**Figure 1A**). Additionally, nodular high-density shadows with poorly defined borders were observed in the left lung (**Figure 1B**). Three days later, a CT-guided lung biopsy was performed under local anesthesia. A chest X-ray taken the following day revealed no evidence of pneumothorax or hydrothorax. Subsequent pathological examination of the right upper lung mass biopsy revealed lung tissue with atypical epithelial cell nests in some areas. Immunohistochemistry confirmed squamous cell carcinoma (**Figures 2A, C**). Immunohistochemistry and oncogene testing results were as follows: CK7 (−), TTF-1 (−), Napsin A (−), CK5/6 (+), P40 (+) (**Figure 2B**), P63 (+), CgA (−), Syn (−), CD56 (−), P53 (mutant, loss of expression), Ki-67 (+, 25%), and PCK (+). The tumor genetic testing report from our hospital’s Precision Medicine Diagnosis Center showed that the tumor was EGFR mutation-negative and had a PD-L1 immunohistochemistry Tumor Proportion Score (TPS) of 60% (**Figure 2D**). Staging was cT4N3M1a, stage IVa. The patient had no history of hypertension, coronary heart disease, or other cardiovascular diseases before chemotherapy and had a normal electrocardiogram.

### 2.2 First-line treatment

Two weeks following the diagnosis of lung cancer, first-line therapy with tislelizumab (200 mg, d1), paclitaxel (180 mg, d1), and carboplatin (300 mg, d2) was initiated on May 31, 2022. After the first cycle, the patient achieved a partial response, as evidenced by significant tumor shrinkage on repeat CT imaging (**Figures 1C, D**). However, the patient subsequently developed hydropneumothorax and, critically, grade 3 immune-mediated myocarditis, confirmed by a marked elevation of cardiac enzymes (CK, CK-MB, and high-sensitivity troponin). Immunotherapy was permanently discontinued.

### 2.3 Complications of hydropneumothorax

**Figure 3:**
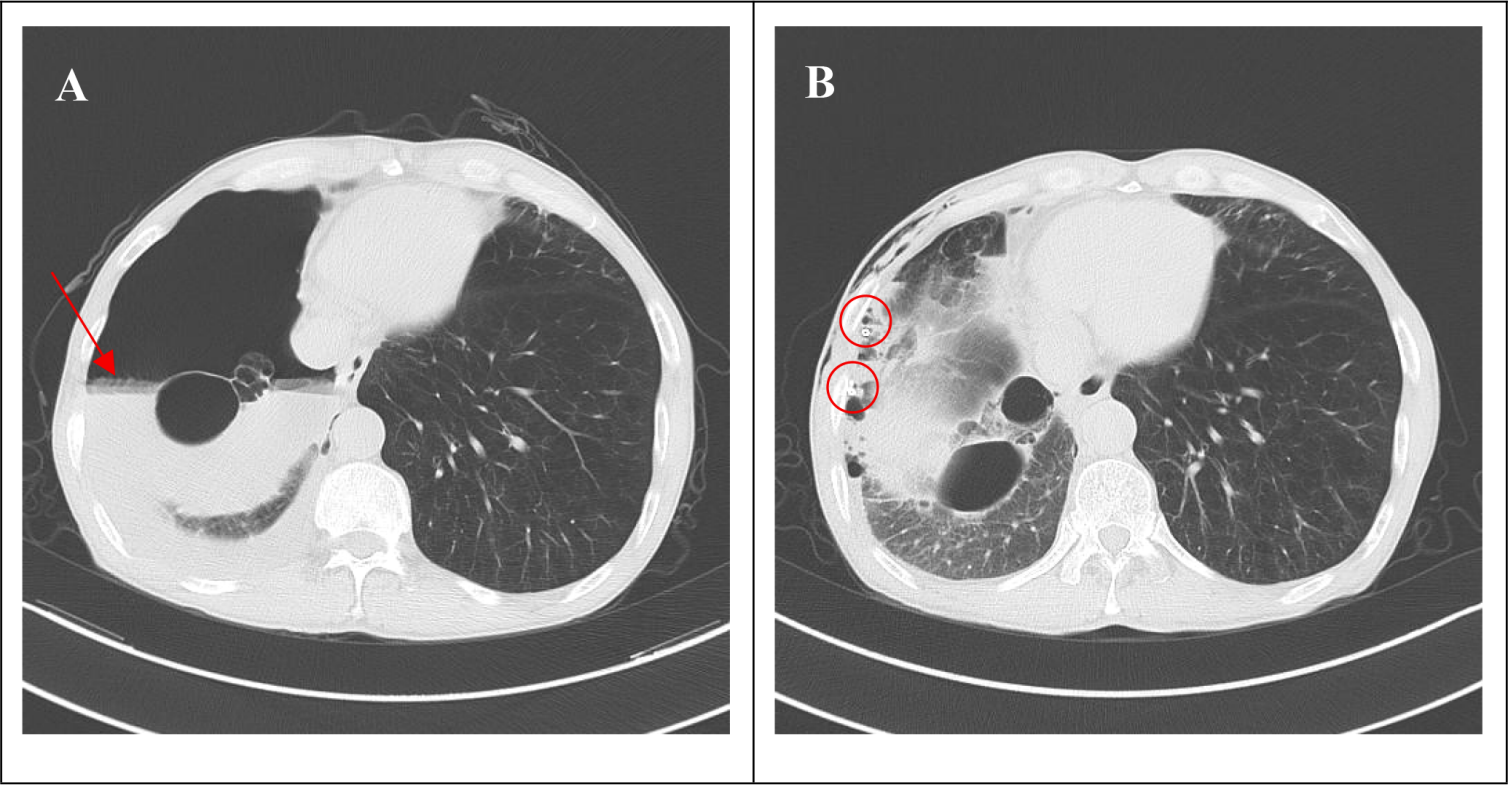
Imaging of hydropneumothorax. **(A)** Chest CT on June 18, 2022 (before treatment for hydropneumothorax): right-sided pneumothorax, pleural effusion, and an air-fluid level (arrow). **(B)** Chest CT on June 21, 2022 (after treatment for hydropneumothorax): two chest drainage tubes in place (circle), showing near-complete resolution of pleural gas and fluid, with good lung re-expansion.

The patient had obstructive pneumonia and pulmonary bullae in the right upper lobe. Eleven days after receiving immunotherapy combined with chemotherapy, the patient experienced increased cough, sputum production, chest tightness, and shortness of breath at home. Three days later, he presented to the hospital. Blood tests after admission revealed no significant abnormalities in creatine kinase (CK), CK-MB, or high-sensitivity troponin. C-reactive protein was elevated at 42.7 mg/L. Obstructive pneumonia had worsened. Treatment included intravenous cefoperazone–sulbactam 2 g q8h for anti-infection therapy, and nebulized acetylcysteine (Fulushi) with salbutamol sulfate for cough and expectoration. Sputum production decreased; however, the patient’s chest tightness and shortness of breath persisted. Four days later, a chest CT scan (**Figure 3A**) revealed hydropneumothorax. After immediate cardiothoracic consultation, closed thoracic drainage was performed. Repeat chest CT conducted three days later (**Figure 3B**) showed near-complete resolution of pleural gas and fluid, with good lung re-expansion. Four days later, the thoracic drainage tube was removed.

A chest X-ray performed one day post-biopsy ruled out immediate complications (e.g., hydropneumothorax). Hydropneumothorax occurred 18 days after starting immunotherapy and chemotherapy. Given the delayed onset (>24 h post-biopsy), concurrent therapy-related pulmonary toxicity, and underlying severe emphysema, the hydropneumothorax was unlikely to be biopsy-related. It was attributed to: 1) treatment-induced tumor necrosis; rapid necrosis after immunotherapy/chemotherapy, especially with bronchial wall invasion, likely caused a bronchopleural fistula and hydropneumothorax, supported by CT findings of tumor shrinkage and resolved obstructive pneumonia; and 2) spontaneous rupture due to emphysema; emphysema increases tissue fragility, and tumor-induced weakness and coughing may trigger bullae rupture, causing pneumothorax, with infection contributing to fluid accumulation.

### 2.4 Complications of immune-mediated myocarditis

**Figure 4:**
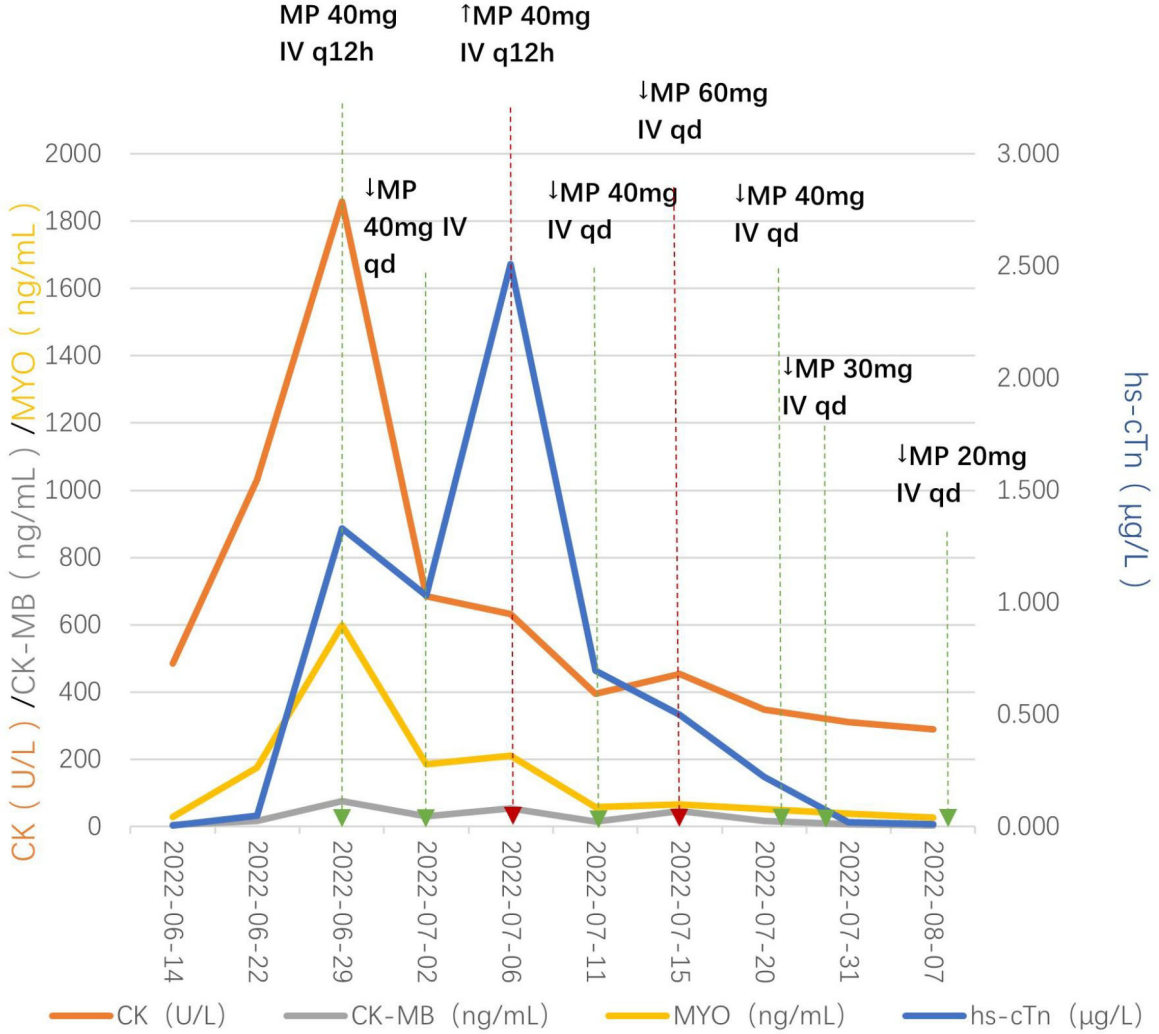
Timeline of dynamic changes in the biochemical indicators of immune myocarditis and response to hormone therapy. Treatment was initiated on June 29, 2022, with intravenous methylprednisolone 40 mg q12h for anti-inflammatory therapy. After 3 days (July 2, 2022), the dose was reduced to 40 mg qd. On July 6, 2022, owing to an increase in CK-MB, the dose was increased to 40 mg q12h. After 5 days (July 11, 2022), the dose was subsequently reduced to 40 mg qd. On July 15, 2022, owing to another rise in CK-MB, the dose was again increased to 60 mg qd. After 7 days (July 22, 2022), it was again reduced to 40 mg qd; after another 7 days (July 29, 2022), it was reduced to 30 mg qd; after 10 days (August 8, 2022), it was reduced to 20 mg qd. On August 9, 2022, the patient’s symptoms improved, and he requested to be discharged. Oral methylprednisolone 20 mg qd was prescribed. The dose was tapered to 16 mg qd after two weeks, reduced to 12 mg qd after another two weeks, reduced to 8 mg qd after another two weeks, reduced to 4 mg qd after another two weeks, reduced to 2 mg qd after another two weeks, and discontinued after a further two weeks.

On the 22nd day after starting immunotherapy, the patient continued to experience chest tightness despite closed thoracic drainage. Serial cardiac monitoring revealed a progressive and significant elevation of cardiac enzymes: CK peaked at 1032 U/L (5x ULN), CK-MB at 16.04 ng/mL (2x ULN), and high-sensitivity troponin I at 0.05 μg/L (2x ULN). Electrocardiography revealed slight T-wave changes, while transthoracic echocardiography showed a preserved left ventricular ejection fraction (LVEF) of 57%. Tislelizumab was consequently permanently discontinued. The patient was instructed to remain on bed rest and receive oxygen therapy. One week later, CK peaked at 1859 U/L (9x ULN), CK-MB at 75.94 ng/mL (15x ULN), and high-sensitivity troponin I at 1.31 μg/L (77x ULN). A diagnosis of grade 3 immune checkpoint inhibitor-related myocarditis was consequently established. Management of the myocarditis involved a multidisciplinary team, including medical oncology, cardiology, cardiothoracic surgery, and clinical pharmacy. Medical oncology was responsible for chemotherapy and immunotherapy decisions; cardiology assessed cardiac enzyme levels and electrocardiographic changes; cardiothoracic surgery performed chest tube drainage for pleural effusion; and clinical pharmacy recommended the corticosteroid dosing regimen. On June 29, 2022, intravenous methylprednisolone 40 mg q12h was initiated. The dose was adjusted based on CK-MB levels: reduced to 40 mg qd after 3 days; increased to 40 mg q12h after 4 days due to CK-MB elevation; increased to 60 mg qd after 5 days; then tapered stepwise as follows: 40 mg qd for 7 days, 30 mg qd for 7 days, and 20 mg qd for 10 days. After symptom improvement, the patient was discharged on oral methylprednisolone 20 mg qd, which was tapered biweekly (16, 12, 8, 4, 2 mg) and then discontinued. The timeline of the diagnosis and treatment process is presented in **Figure 4**.

### 2.5 Follow up multi-line treatment and mortality outcomes

**Figure 5:**
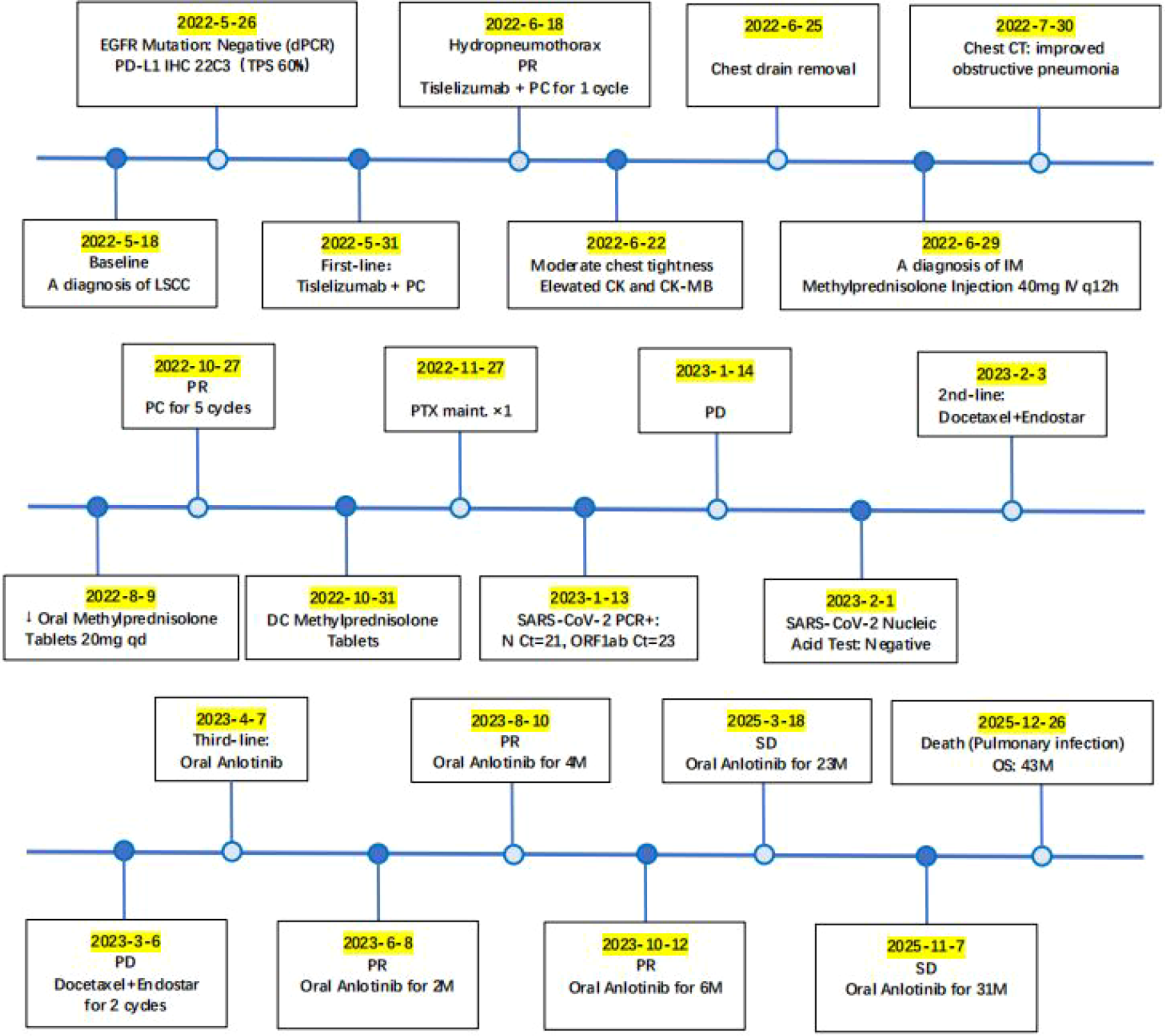
Timeline of the patient’s therapeutic management. This timeline depicts the clinical course of the patient with advanced lung squamous cell carcinoma (PD-L1 TPS 60%, EGFR negative). First-line therapy with tislelizumab plus chemotherapy was complicated by immune-mediated myositis, which was effectively managed with corticosteroids. After achieving an initial partial response (PR) followed by subsequent progression, the patient received second-line therapy followed by third-line anlotinib, which resulted in a prolonged partial response and sustained disease stabilization for over 31 months. Unfortunately, the patient ultimately died from a pulmonary infection. The total survival period was 43 months.

**Figure 6:**
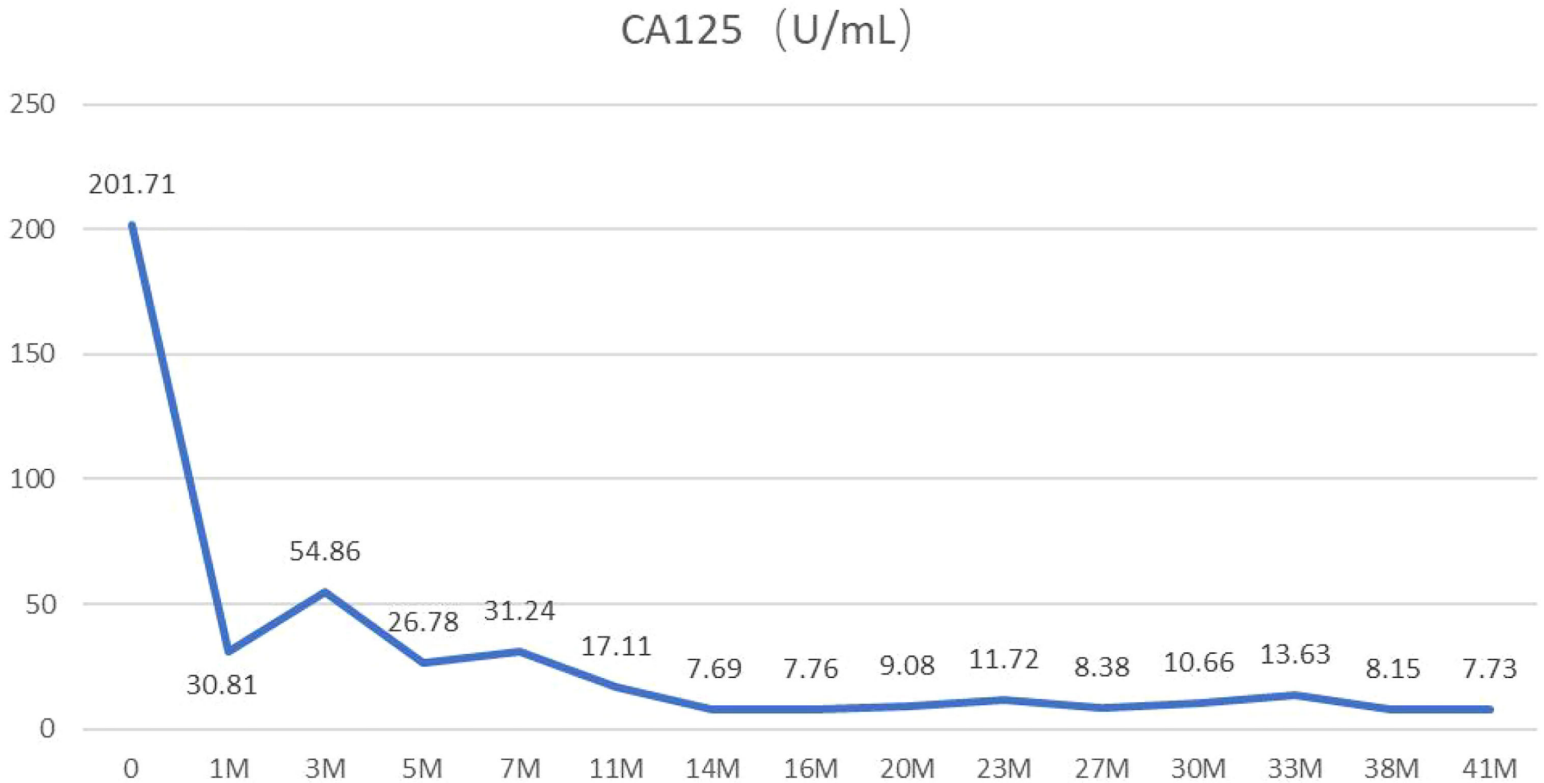
Serial measurements of CA125 levels. At diagnosis, the patient’s CA125 level was 201.71 U/mL. After treatment, this level decreased significantly. More than a month before death, it was measured at 7.73 U/mL, which is within the normal range.

Subsequently, the patient received five additional cycles of chemotherapy (paclitaxel/carboplatin) alone, maintaining stable disease (**Figures 1E, F**) until radiographic progression (**Figures 1G, H**) was noted following a mild SARS-CoV-2 infection. Second-line therapy with docetaxel and the anti-angiogenic agent Endostar was initiated but was complicated by hemoptysis, leading to discontinuation of Endostar. Upon further disease progression (**Figures 1I, J**), third-line therapy with the multi-target tyrosine kinase inhibitor (TKI) anlotinib (10 mg, d1–14, q3w) was initiated. This intervention resulted in a sustained partial response (**Figures 1K, L**), with the patient maintaining a progression-free period of 31 months at the last follow-up (**Figures 1M, N**) and an OS of 43 months (**Figure 5**). Over a month before the patient’s death, a chest CT scan showed stable disease, with tumor markers within the normal range (**Figure 6**), indicating no disease progression. Follow-up communication with the patient’s family revealed that the patient developed fever, increased cough and sputum production, chest tightness, and shortness of breath at home. The patient died of pneumonia.

## 3 Discussion

In a case of PD-L1-high squamous NSCLC with grade 3 cardiotoxicity, salvage anlotinib provided durable disease control, suggesting that VEGF inhibition may be a post-ICI strategy. Grade 3 immune-related cardiotoxicity mandates immediate ICI withdrawal. Salvage anlotinib plus sequential chemotherapy, followed by maintenance antiangiogenic therapy, may achieve long-term survival in PD-L1-high squamous NSCLC.

### 3.1 Myocarditis: diagnosis and differential

In this patient, transthoracic echocardiography revealed no pericardial effusion or cardiac mass, effectively ruling out cardiac tumors. The LVEF was 57%, excluding heart failure. Asymptomatic valvular thickening was noted, with no evidence of valvular disease. Electrocardiography demonstrated sinus rhythm with non-specific T-wave changes, ruling out atrial fibrillation, atrial flutter, and atrioventricular block. The D-dimer level was 2.48 mg/L, making pulmonary embolism unlikely. In the absence of chest pain and with unremarkable electrocardiographic findings, myocardial infarction was excluded. The patient presented with dyspnea and chest tightness that persisted despite pleural drainage and was unresponsive to oxygen therapy. Symptoms were severe even at rest, necessitating active intervention. Given the history of ICI use and elevated levels of CK, CK-MB, and high-sensitivity troponin I, the patient was diagnosed with immune-related myocarditis. According to the Common Terminology Criteria for Adverse Events (CTCAE) version 5.0, the severity was classified as grade 3.

### 3.2 Treatment selection

#### 3.2.1 Selection of first-line therapy

At initial diagnosis, the patient has cT4N3M1a stage IVa squamous cell carcinoma, no driver mutations, EGFR wild-type, and a PD-L1 TPS of 60%. According to the 2021 Chinese Society of Clinical Oncology (CSCO) guidelines, pembrolizumab monotherapy (Category I) is indicated for patients with PS 0–1 and PD-L1 TPS ≥50% (13). As pembrolizumab was not covered by insurance, the patient opted for chemotherapy plus immunotherapy (also Category I): paclitaxel + carboplatin + tislelizumab. Tislelizumab, a PD-1 inhibitor, blocks PD-1/PD-L1 binding to activate antitumor immunity (15, 16). The RATIONALE-307 trial showed that adding tislelizumab to chemotherapy prolonged PFS (9.6 vs. 5.5 months). Treatment was discontinued due to immune-mediated myocarditis. The patient then received five cycles of chemotherapy alone (paclitaxel + carboplatin). After six cycles, the disease remained stable, and maintenance paclitaxel was initiated.

#### 3.2.2 Selection of second-line therapy

Upon disease progression, second-line therapy was required for stage IV driver-negative squamous NSCLC. The 2022 CSCO guidelines recommend docetaxel (Category I) for patients with PS 0–2 (17). Docetaxel is an antimicrotubule agent that inhibits mitosis (18). The TAX320 trial reported an objective response rate (ORR) of 10.8% with docetaxel compared with comparators. Given the modest ORR of docetaxel and prior discontinuation of immunotherapy, Endostar (recombinant human endostatin), an antiangiogenic agent, was added (19). Endostar was later discontinued due to hemoptysis.

#### 3.2.3 Selection of third-line therapy

After further progression, third-line therapy was initiated. The 2022 CSCO guidelines recommend anlotinib (Category II, level IB evidence) for patients with PS 0–2 (17). Anlotinib is a multi-target TKI targeting VEGFR, PDGFR, and FGFR (18). The ALTER0303 trial demonstrated that anlotinib improved PFS and OS in advanced NSCLC after failure of second-line chemotherapy (20). In this case, PFS with anlotinib reached 31 months, far exceeding the reported median PFS of 5.6 months (21). At the last follow-up, the patient died of a pulmonary infection, with a final OS of 43 months.

### 3.3 Management of immune-mediated myocarditis

The exact mechanisms underlying immune checkpoint inhibitor-induced myocarditis remain incompletely understood. One consistent pathological observation in affected patients is the prominent accumulation of pro-inflammatory CD3+ T cells—predominantly cytotoxic CD8+ T cells, which outnumber CD4+ T cells—alongside CD68+ macrophages within the myocardial tissue. The infiltration of these immune cells leads to cardiomyocyte injury and necrosis (22–24). Risk factors for immune-related myocarditis include advanced age, male sex, a history of cardiovascular disease, smoking, high body mass index, lung cancer, pre-existing autoimmune disease, absence of corticosteroid use, combination immunotherapy, and radiotherapy, among others (25–27). Furthermore, the clinical characteristics of irAEs may vary depending on the specific ICI used. Following the approval of the first CTLA-4 inhibitor, ipilimumab, for metastatic melanoma in 2011, an increasing number of ICIs have been approved for a broad spectrum of malignancies, including lung cancer, breast cancer, head and neck cancer, genitourinary cancers, gastrointestinal cancers, and gynecologic malignancies. These agents include another CTLA-4 inhibitor (tremelimumab), PD-1 inhibitors (nivolumab, toripalimab, pembrolizumab, and cemiplimab), and PD-L1 inhibitors (durvalumab, atezolizumab, and avelumab). More recently, agents targeting lymphocyte activation gene-3 (LAG-3), such as relatlimab, have also been introduced (28). Furthermore, ICIs—such as CTLA-4/PD-1 inhibitors and PD-1/LAG-3 inhibitors—are increasingly used in combination regimens, which are associated with an elevated risk of irAEs. In contrast, the management of immune-related myocarditis is relatively well-established.

Once a diagnosis of immune-related myocarditis is established, the implicated immune checkpoint inhibitor must be immediately discontinued. Corticosteroids are recommended as first-line treatment by both domestic and international guidelines (29–32). The standard treatment approach involves the prompt initiation of high-dose corticosteroid pulse therapy—typically 500–1000 mg daily for three to five days—followed by a scheduled gradual tapering over four to six weeks. During the tapering period, close monitoring of patient-reported symptoms, electrocardiographic parameters, and serial troponin measurements is essential to allow individualized treatment adjustments based on clinical response. For patients who show an inadequate response to corticosteroids or have persistently elevated troponin levels, combination therapy with additional immunomodulatory agents is generally considered. Various second-line therapeutic options have been reported, including T cell-directed agents (e.g., tacrolimus), cytokine-modulating therapies (e.g., ruxolitinib), antibody-based interventions (e.g., intravenous immunoglobulin), and monoclonal antibodies such as abatacept and alemtuzumab (33–44). Concurrently, ongoing scientific investigations continue to explore additional therapeutic possibilities (37).

In this patient, tislelizumab (a PD-1 inhibitor) likely triggered T-cell overactivation against shared antigens on cardiomyocytes, causing inflammatory damage (45). Immediate discontinuation at initial CK elevation (G3 toxicity) aligned with the 2021 CSCO Guidelines for the Management of Immune Checkpoint Inhibitor-Related Toxicities, which recommend permanent ICI discontinuation for G3 myocarditis. Multidisciplinary consultation (cardiothoracic surgery andcardiology) guided rapid glucocorticoid initiation (46, 47). Methylprednisolone [initial dose 1–4 mg/(kg·d)] was administered for 3–5 days and then gradually tapered. Dose adjustments based on CK-MB rebound reflected dynamic, individualized management. A stepwise oral taper (reducing by 4 mg every 2 weeks) minimized relapse risk, enabling discontinuation after nearly 3 months of intravenous-to-oral sequential therapy.

Immune-related myocarditis typically presents with rapid onset, poor prognosis, and low salvage rates; only a few successful cases have been reported. Two patients with metastatic melanoma developed fulminant myocarditis after nivolumab and ipilimumab, respectively, and both died (9). The successful management in this case provides a reference for future early warning and therapeutic strategies.

### 3.4 The role of anlotinib in the tumor microenvironment

The exceptional survival achieved in this patient after discontinuation of immunotherapy is the most striking finding. An initial tumor response followed a single cycle of an immune checkpoint inhibitor. The disease subsequently progressed during maintenance chemotherapy. Second-line therapy with docetaxel and Endostar also failed. Finally, a profound and durable response was achieved with third-line anlotinib. Based on these findings, we propose a two-phase model involving immune priming and subsequent microenvironment maintenance. A single dose of tislelizumab likely provided the necessary “priming” event. It may have durably activated tumor-specific T-cell clones. Conventional chemotherapy lacks immunomodulatory activity and fails to sustain this effect.

Unlike Endostar, anlotinib is a multifaceted modulator of the tumor immune microenvironment (48). Endostar exerts a purely anti-angiogenic effect, which may paradoxically exacerbate immunosuppression by disrupting the vasculature and creating hypoxia (49). In contrast, anlotinib promotes vascular normalization, a process known to improve T-cell infiltration into tumors (50). In addition, anlotinib may enhance the efficacy of anti-PD-1 therapy through dual mechanisms: by inhibiting the AKT pathway and by promoting apoptosis of cancer-associated fibroblasts in lung adenocarcinoma (51). In the RATIONALE-307 trial (52), the combination of tislelizumab and chemotherapy achieved a median OS of 22.8 months, compared with 20.2 months with chemotherapy alone, representing a 2.6-month improvement. After resolution of immune-related cardiotoxicity, the patient commenced oral anlotinib therapy, achieving a PFS of 31 months and an OS of 43 months. This represents a favorable outcome, with overall survival substantially exceeding the median OS reported in the RATIONALE-307 trial.

### 3.5 Limitations

This case report shares the inherent limitations of all such studies, including high selectivity and potential bias, as it focuses on a rare, atypical, or representative individual case that cannot be generalized. Moreover, the patient died at home without an autopsy; therefore, the cause of death was based on family−reported symptoms. While high fever, cough, and sputum are highly suggestive of infectious pneumonia, immune-related pneumonia or drug-induced lung injury cannot be ruled out. We emphasize that, in patients receiving immune checkpoint inhibitors and anti-angiogenic agents who develop respiratory symptoms, bronchoscopy, pathogen testing, and HRCT should be prioritized to determine the etiology. This case highlights that even in advanced disease, obtaining etiological evidence is essential for treatment guidance and prognosis.

## 4 Conclusion

Overall, this case demonstrates the following: prompt ICI discontinuation and cardiac support are critical for the management of severe irAEs, and anti-angiogenic therapy may provide durable disease control after failure of immunotherapy. In the present patient with advanced lung squamous cell carcinoma complicated by pulmonary bullae, after experiencing hydropneumothorax, immune-related myocarditis, and SARS-CoV-2 infection, treatment with anlotinib achieved disease stabilization for 31 months, with a total survival of 43 months. This successful outcome was achieved through a multidisciplinary team approach involving individuals with expertise in oncology, cardiothoracic surgery, cardiology, and clinical pharmacy.

## Data Availability

The datasets presented in this study can be found in online repositories. The names of the repository/repositories and accession number(s) can be found in the article/supplementary material.
